# Questionnaires that measure the quality of relationships between patients and primary care providers: a systematic review

**DOI:** 10.1186/s12913-018-3687-4

**Published:** 2018-11-19

**Authors:** Lauren E. Ball, Katelyn A. Barnes, Lisa Crossland, Caroline Nicholson, Claire Jackson

**Affiliations:** 1 Centre for Health System Reform and Integration, UQ-Mater Research Institute, Brisbane, Australia; 2 Menzies Health Institute Queensland, Griffith University, Parklands Drive, Southport, Gold Coast, QLD 4222 Australia

**Keywords:** Continuity of care, General practice, Physician-patient relations, Primary care, Relationships, Survey, Therapeutic Alliance, Questionnaire

## Abstract

**Background:**

International guidance on models of care stress the importance of good quality, continuous patient-provider relationships to support high quality and efficient care and hospital avoidance. However, assessing the quality of patient-provider relationships is challenging due to its experiential nature. The aim of this study was to undertake a systematic review to identify questionnaires previously developed or used to assess the quality of continuous relationships between patients and their provider in primary care.

**Methods:**

MEDLINE, PubMed, Cumulative Index of Nursing and Allied Health Literature (CINAHL) and SCOPUS databases were searched for English language studies published between 2009 and 2017. Key terms used identified studies conducted in the primary care setting examining relationships between patients and providers. Studies that focused on the conceptualisation, development, testing or review of a questionnaire, or studies that used a questionnaire for assessing the quality of continuous relationships between patients and providers were eligible. Studies that did not assess quality via a questionnaire, only assessed single aspects of relationships, only assessed single encounters, assessed transitions between settings or assessed relationships using an index were excluded. Information on validity testing of each relevant questionnaire identified from articles was reviewed to inform recommendations for future research and evaluation.

**Results:**

Twenty-seven studies met the eligibility criteria, including 14 unique questionnaires. The questionnaires were diverse in length, scope, focus and level of validity testing. Five questionnaires were considered not feasible for future use due to size and lack of development work. Three questionnaires were considered strongest candidates for use in future work based on being relevant to the topic and primary care setting, freely available in English and not needing additional pilot work prior to use. These three questionnaires were the Care Continuity Across Levels of Care Scale, the Nijmegan Continuity Questionnaire and the Patient-Doctor Depth of Relationship Tool.

**Conclusions:**

This study provides an overview of 14 unique questionnaires that have been used to assess the quality of continuous relationships between patients and primary care providers. The decision to use one of the questionnaires in future work requires careful consideration, including the scope, length, validation testing, accessibility of the questionnaires and their alignment with the initiative being evaluated.

## Background

The focus of primary care is changing in many countries, with the aging of populations and growing need for continuity rather than episodic care. Structural reforms such as the ‘Patient-Centered Medical Home’ (North America) and ‘Health Care Home’ (Australia & New Zealand) are helping services move away from transaction-based care towards care that is patient-centred and continuous [1, 2]. This change in focus emphasises the importance of all patients having a high-quality relationship with a primary care provider that continues over time [2]. ‘Continuity of Care’ has seemingly been associated with improved clinical outcomes, but the critical elements in play remain undescribed. Concepts such as therapeutic alliance [3], working alliance [4], continuity of care [5], relational continuity [6] and relationship-based care [7] describe the positive outcomes that occur when a patient has a sense of affiliation, collaboration and trust with a single provider that is ongoing in nature [8]. These high-quality relationships have been shown to result in positive patient experiences, greater patient satisfaction, increased treatment adherence and improved patient outcomes [3, 4, 9]. Supporting these continuous, high-quality relationships is clearly warranted.

Assessing the quality of relationship between patients and providers is challenging due to its experiential nature. There is no universal agreement about the definition of quality relationships or the components that underpin the concept, making it challenging to develop valid and reliable assessment tools (questionnaires). Furthermore, the quality of relationships between patients and providers is thought to be influenced by demographic factors of the patient and provider, role of medical receptionists and other staff, and organisational factors of general practice clinics [5]. It is therefore not surprising that quality of relationship is one of the least commonly evaluated aspects of care and there is no recommendation on how to evaluate relationship quality within the reforms happening to general practice [10, 11].

A systematic review has previously been conducted to identify questionnaires that can be used to assess the quality of relationships between patients and doctors across all health care settings [12]. The search was conducted in 2009 and nineteen tools were identified, with variable levels of validity testing to support their development. The review methodology provided a wide reach of measures to consider, but none of the questionnaires were developed for use in the primary care setting where the majority of patients and families experience ongoing care. As a result, there is still no best approach recommended for primary care and the feasibility of these reviewed measures, whilst important, is unknown.

The aim of this study was to conduct a systematic review of the body of evidence for studies that measure the quality of continuous relationships between patients and primary care providers. The review will identify questionnaires developed or used since the previous systematic review [12] and will also appraise the questionnaires on their validity and feasibility for use in the primary care setting. The review will inform evaluation strategies for health care homes.

## Methods

### Overview

A systematic review was conducted to identify measures of continuous quality relationships between patients and providers in primary health care. For the purpose of the review, ‘relationships’ referred to an ongoing sense of affiliation and collaboration with a provider in primary care, typically a General Practitioner (GP) [8]. The systematic review was conducted in accordance with the Preferred Reporting Items for Systematic reviews and Meta Analyses (PRISMA) statement [13].

### Literature search

A systematic computer-based literature search was conducted between March and June 2017. Databases searched were MEDLINE, PubMed, Cumulative Index of Nursing and Allied Health Literature (CINAHL) and SCOPUS. Medical subject headings (MeSH), were used in the execution of PubMed and MEDLINE database searches. Boolean connectors AND and OR were used to combine search terms. Three categories of search terms were used; (i) terms relating to the setting: ‘primary care’, ‘primary health care’ and ‘general practice’, (ii) terms relating to relationships: ‘relational continuity’, ‘continuity of care’, ‘physician-patient relations’, ‘professional-patient relations’, ‘therapeutic alliance’, ‘patient participation’, and ‘patient empowerment’, and (iii) terms relating to the methodological focus of the study: ‘tool’, ‘instrument’, ‘scale’, ‘survey’, ‘questionnaire’ and ‘measure’. Google Scholar and PUBMED were used to obtain additional articles identified by journal hand searching. All database search results were imported into EndNote and duplicates removed prior to screening.

### Eligibility criteria

Studies were included in the review if: 1) they focused on the conceptualisation, development, testing or review of a questionnaire for measuring the quality of continuous relationships between patients and a primary care provider; or 2) they used a questionnaire for assessing the quality of continuous relationships between patients and a primary care provider. Studies were also included if the authors’ interpretation of “relationships” related to patients having a sense of affiliation, collaboration and trust with a single provider that is ongoing in nature, including phrases such as therapeutic alliance, working alliance, continuity of care and relational continuity. All study designs were considered relevant, including observational, descriptive, intervention and theoretical methodologies. Studies needed to be available in full-text, English and published between the years 2009–2017. This time period was chosen because the literature search in the previous systematic review related to this topic occurred in 2009 [12]. The focus on health care homes as an approach to primary health care reform has also occurred since this time [1].

Studies were excluded if they described the importance of high quality relationships without measuring or assessing these (i.e. via a questionnaire). All remaining studies that utilised a questionnaire were excluded if the questionnaire assessed: (1) single encounters only (rather than continuous care), (2) single aspects of relationships (such as communication), (3) transitions between health care settings (such as attending primary care after hospital discharge; informational continuity) or (4) assessed the quality of relationships *between providers* in a multidisciplinary team. Finally, studies that used a formulaic index to assess relationships (such as the number of different providers seen in a year) were also excluded due to the inability to assess the quality of relationships using this approach.

### Study selection

**Fig. 1:**
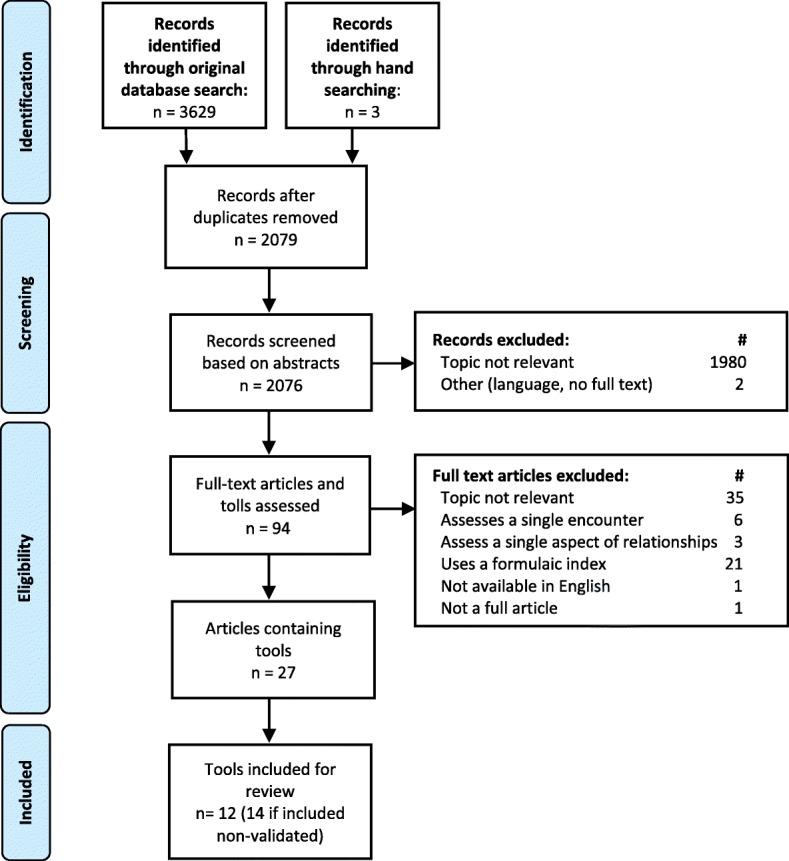
PRISMA diagram of the literature search and filtering results for a systematic review of the questionnaires used to measure continuous relationships in primary care

The study selection process is illustrated in Fig. 1. A quality control training procedure was conducted to ensure consistency of coding between reviewers. Three reviewers independently read the abstracts of the first 100 articles identified in the search and coded them as ‘retrieve full text’ if the article met the inclusion criteria; ‘exclude’ if the article did not meet the eligibility criteria or ‘unsure’ if the reviewer was not able to make a decision. Agreement between all reviewers was obtained for 62/100 abstracts (62%), and at least one reviewer coded ‘unsure’ for the remainder of articles. Where the coding differed, consensus was achieved through group discussion. Another 50 abstracts were then reviewed and coded independently, with agreement for 46/50 (92%) abstracts obtained. Following another group discussion, the remaining abstracts were divided between the three reviewers for independent, duplicate coding.

Full manuscripts were retrieved for those studies coded by two reviewers to meet the inclusion criteria or where more information was required in order to make a decision. Disagreements between duplicate reviewers were considered by the third reviewer and resolved via group discussion. Reference lists from all systematic review articles retrieved but not included were cross-checked to identify additional articles not captured in the original search. These studies were subjected to identical abstract review.

### Data extraction

Data from all included articles were extracted using an electronic spread sheet developed specifically for this review. Information extracted included authorship team; year of publication; country; stated aim; participant characteristics (age, sex and relevant health conditions); phrases used to indicate its relevance to the review topic; and relevant tools used in the study. For each questionnaire, information about the name, author, number of items, answer format, target respondents and validation activities were extracted into a separate electronic spread sheet. Hand searching was conducted on each questionnaire to identify information not provided in the reviewed article, including validation work.

### Risk of bias and data analysis

Quality assessment of included articles was not undertaken for this review as it does not draw conclusions from the findings of the articles. Rather, information on validity testing of questionnaires was extracted, covering internal consistency, construct validity, test-retest reliability, face validity, and test discriminate validity. This information was analysed by two reviewers using an iterative process of data extraction, discussions and contacting authors of questionnaires where required. Decisions about the appropriateness of questionnaires to primary health care were made in group meetings after considering the focus, length, validation and availability.

## Results

The initial database search identified 3629 articles for screening as outlined in Fig. 1. Within this group, four systematic reviews were screened and although none met the inclusion criteria, their reference lists identified an additional three articles for consideration. After removing duplicate copies of articles, the main reasons for excluding articles were: the topic not being relevant (*n* = 2015); the study using a formulaic index to assess relationships (*n* = 21); the study examining single encounters only (*n* = 6), the study examining single aspects of relationships such as communication (*n* = 3); or the study not being available in full-text in English (*n* = 4). This left 27 studies eligible for inclusion.

Table 1 outlines the characteristics of the 27 studies included in the review. Of the 27 studies, eight studies focused on the conceptualisation, development, testing or review of a questionnaire [14–21] and 19 studies used a questionnaire in a study investigating the quality of relationships between patients and primary care providers [6, 19, 22–38]. The following phrases were used in the studies to refer to “quality of relationships” and considered synonymous with the topic of this review: care continuity, continuing relationship, continuity of care, interpersonal care, long term relationships, longitudinality of care, patient-doctor relationships, patient-provider relationships, personal continuity, quality of care, relational continuity, relations, therapeutic alliance, therapeutic relationship.

**Table 1:** Characteristics of included studies assessing the quality of continuous relationships between patients and providers in primary care grouped by inclusion criteria and in alphabetical order of first author

Author (Year)	Country	Aim of study	Sample (Participants)	Phrases used to indicate “quality relationships”	Type of relevant questionnaire(s)	Name of relevant questionnaire(s)
Studies about the conceptualisation, development, testing, or review of a tool for assessing the quality of relationships between patients and providers in primary care						
Burge et al. (2011) [14]	Canada	To examine how well relational continuity is measured in validated instruments that evaluate primary healthcare from the patient’s perspective.	N/A – Review of validated questionnaire and subscales	Relational continuity Therapeutic relationship	Validated questionnaires	Primary Care Assessment Survey Primary Care Assessment Tool (short form) Components of Primary Care Index
Haggerty et al. (2012) [15]	Canada	To develop and validate a generic measure of management continuity from the patient perspective.	Patients who had received care for an ongoing health condition at more than one clinic in the previous 12 months.	Relational continuity	Questionnaire (being validated)	Generic measure of continuity of care
Harley (2009) [16]	England	To adapt the Components of Primary Care Index (CPCI) to be applicable to oncology outpatients and to assess the reliability and validity of the adapted instrument (renamed the Medical Care Questionnaire [MCQ]).	Outpatient cancer patients ≥18 years of age.	Continuity of care Coordination of care	Questionnaire	Medical Care Questionnaire
Hill et al. (2014) [17]	England	To determine the suitability of the Primary Care Assessment Tool as a measure of continuity of care for patients with a long-term condition (stroke), and co-morbidity, in a primary care setting.	Community living stroke survivors (12 months post stroke)	Continuity of care Relational continuity	Questionnaire	Chao Perception of Continuity Scale
Jatrana (2011) [18]	New Zealand	To construct a summary measure of continuity of care	Randomly sampled individuals, aged ≥15 years, who completed Statistics New Zealand-led Survey of Family, Income and Employment (SoFIE) survey	Continuity of care	4 questions identified from the PCAT	Primary Care Assessment Tool
Uijen (2011) [20]	Netherlands	To develop and pilot test a generic questionnaire to measure continuity of care from the patient’s perspective across primary and secondary care settings.	Patients aged > 18 years, with at least one chronic illness, literate in Dutch.	Patient provider relationship Personal continuity	Questionnaire (being validated)	Nijmegen continuity questionnaire
Uijen (2012) [40]	Netherlands	To further examine the validity, discriminative ability, and reliability of the Nijmegen continuity questionnaire.	Patients aged > 18 years, with at least one chronic illness, literate in Dutch.	Continuity of care Personal continuity Quality of care	Questionnaire (being validated)	Nijmegen continuity questionnaire
Zenger (2014) [21]	Germany	To assess the internal and external validity of the German version of the PDRQ-9 in a representative cross-sectional German population	Randomly selected individuals, ≥14 years of age, literate in German, who had visited a PCP.	Patient-doctor relationship, therapeutic alliance,	Validated questionnaire	Patient Doctor Relationship Questionnaire (PDQR-9)
Studies that use a tool for assessing the quality of relationships between patients and providers in primary care						
Al-Azri et al. (2014) [22]	Oman	To study the role of relational continuity in primary care settings and its effect on patients’ perceptions and experiences.	Patients aged > 18 years attending their PCHCs during the study period	Relational continuity	Non-validated questionnaire	N/A
Bryan et al. (2012) [24]	United States of America	To identify the impact of very early therapeutic alliance on the general trajectory of change for suicidal ideation among patients seen within the context of an integrated primary care behavioural health service.	Patients with mental health concerns, receiving a referral from their primary care provider to an Air-Force hospital based Behavioural Health Consultant.	Therapeutic alliance	Validated questionnaire	Therapeutic Bond Scale
Corso et al. (2012) [25]	United States of America	To investigate therapeutic alliance and clinical improvement within an integrated primary care behavioural health model	Patients with mental health concerns receiving a referral from their primary care provider to an Air-Force hospital based Behavioural Health Consultant.	Therapeutic alliance	Validated questionnaire	Therapeutic Bond Scale
Falkenström et al. (2013) [26]	Sweden	Test whether high alliance scores after a consultation predicts lower symptom scores immediately before the next consultation	Patients aged 18–70 years, attending a health service and receiving treatment with psychologist, social worker or counsellor	Therapeutic alliance	Validated questionnaire	Working Alliance Inventory, short form (revised)
Ferrer et al. (2014) [27]	Brazil	To compare two offered care models in relation to longitudinality care, from the users’ perspective, and to correlate this finding to the utilisation of PHC services among patients hospitalised due to preventable conditions.	Children aged 0–14 years, attending paediatric ward of Sao Paulo hospital during the study period	Longitudinally of care Care continuity	Validated questionnaire	Primary Care Assessment Tool (child version)
Hansen (2016) [28]	Norway	To explore how women with CFS/ME experience GP care regarding informational, management, and relational continuity.	Members of the Norwegian Myalgic Encephalomyelitis association, experiencing Chronic Fatigue Syndrome	Relational continuity	Non-validated questionnaire	N/A
Hernandez, A. et al. (2013)a [29]	Spain / Catalonia	To determine patients’ perceived degree of continuity of care between primary and secondary care and to identify contextual and individual factors that influence patients’ perceptions of continuity of care.	Patients ≥18 years of age who had received primary and secondary care in Catalonia within the previous 3 months.	Relational continuity	Validated Questionnaire	Care Continuity Across Levels of Care Scale (CCAENA)
Hernandez, A. et al. (2013)b [30]	Spain / Catalonia	To compare immigrants’ and natives’ perceptions of relational, managerial and informational continuity of care and to explore the influence of the length of stay on perceptions of continuity.	Patients ≥18 years of age who had received primary and secondary care in Catalonia within the previous 3 months.	Relational continuity	Validated Questionnaire	Care Continuity Across Levels of Care Scale (CCAENA)
Hernandez, A. et al. (2013)c [31]	Spain / Catalonia	To provide additional evidence on the psychometric properties the scales of this questionnaire.	Patients ≥18 years of age who had received primary and secondary care in Catalonia within the previous 3 months.	Patient–primary care provider relationship, Continuity across care	Validated Questionnaire	Care Continuity Across Levels of Care Scale (CCAENA)
Hernandez, A. et al. (2013)d [31]	Spain / Catalonia	To analyse patient’s reported elements of relational, informational and managerial (dis)continuity between primary and outpatient secondary care and to identify associated factors.	Patients ≥18 years of age who had received primary and secondary care in Catalonia within the previous 3 months.	Relational (dis)continuity	Validated Questionnaire	Care Continuity Across Levels of Care Scale (CCAENA)
Hernandez, SE. et al. (2016) [33]	United States of America	To estimate if the degree of PACT (Patient Alignment Care Teams) implementation at a facility varied with the percentage of minority veteran patients at the facility.	Primary care facilities and	Continuity of care	Validated Questionnaire	Primary Care Assessment Tool (Pi2 – provider tool)
Jahromi (2017) [34]	Iran	To determine the continuity of health care in urban health centres in Iran	Patients and family physicians from participating primary care centres	Interpersonal continuity of care	Validated questionnaire	Primary Care Evaluation Tool (PCET)
Kristjansson (2013) [6]	Canada	To assess whether there was a difference in the continuity of care provided by different models of primary care	Health professionals and patients ≥18 years of age, cognitively intact and not acutely ill	Continuity of care Relational continuity	Validated questionnaire	Primary Care Assessment Tool (PCAT)
Liu (2017) [35]	China	To understand the relationship preferences of primary care patients and their associations with patient experience of continuity of care.	Patients aged ≥18 years attending a community health clinic in Beijing, and not acutely ill.	Continuity of care Relational continuity Continuing relationship	Validated questionnaire	Care Continuity Across Levels of Care Scale (CCAENA)
Merriel (2015) [36]	United Kingdom	To assess whether differences in the depth of relationship between a patient and their GP affects the length of consultations, and the number and type of problems and issues raised during a consultation.	Patients aged ≥18 years with a PHP appointment at a participating primary care clinic.	Patient-doctor continuity Patient-doctor relationship	Validated questionnaire	Patient-Doctor Depth of Relationship
Uijen (2012) [19]	Netherlands	To explore heart failure patients’ experiences with continuity of care, and its relation to medication adherence.	Primary care patients with chronic heart failure, literate in Dutch, no terminal diagnosis, and no mental impairment.	Continuity of care Personal continuity	Non-validated Questionnaire	N/A
Uijen (2014) [37]	Netherlands	To explore the level of experienced continuity of care of patients at risk for depression in primary care, and compare these to those of patients with heart failure	Patients with diagnosed depression or heart failure within the last 12 months, literate in Dutch, no terminal diagnosis, and no mental impairment.	Continuity of care Personal continuity	Questionnaire (adapted abut not re-validated)	Nijmegen Continuity Questionnaire (Brief version)
Wei (2015) [38]	China	To assess changes in the quality of primary care in two megacities following the introduction of health system reforms in China.	Patients aged ≥18 years, attending community health centres in Shenzhen, or Shanghai	Long-term relationships between patients and general practitioners Continuity	Questionnaire (adapted but not re-validated)	Primary Care Assessment Tool (Chinese translation)

*N/A* Not applicable

Some questionnaires were used in several of the studies. Therefore, although 27 studies were included in the review, only 14 questionnaires were used. Table 2 summarises the 14 relevant questionnaires used in the studies. Three of the questionnaires (Primary Care Assessment Survey; Primary Care Assessment Tool; Primary Care Evaluation Tool) were large instruments investigating multiple components of quality care, with only a very small section (e.g. one subscale) examining quality of relationships. These questionnaires were considered unfeasible for future use because most of the data would be irrelevant to the topic. Also, they would require substantial time (e.g. 45 min for the Primary Care Assessment Survey) to complete the questionnaire. Two of the questionnaires (neither with a name) were developed only for use in the reviewed study and were not pilot tested for the purpose of others’ utilising the questionnaires in work [22, 28]. These two questionnaires were considered unfeasible for future use as there was no evidence to support their validity. This left nine questionnaires that were examined further.

**Table 2:** Description of questionnaires used in studies to assess quality of relationships between patients and primary care providers in alphabetical order

Questionnaire	Authors of the questionnaire or validation paper(s)	Questionnaire Description	Questionnaire Format	Answer format	Summary of validation work
Care Continuity Across Levels of Care Scale (CCAENA)	Herndandez, A (2010)	Aims to assess patients’ perspectives of assess care continuity across settings	138 items across three subscales: 1) Pt-PCP relational continuity^a^ 2) Pt-Secondary Care provider relational continuity3) Transfer of information	6 point Likert scale on level of agreement with statements; 2 open ended	Pilot study on 1500 patients to confirm construct validity against predetermined subscales (all had eigenvalues greater than 1) and confirm internal consistency (Cronbach’s alpha 0.8–0.97) [31].
Chao Perception of Continuity Scale	Chao (1988)	Aims to assess patients’ perception of continuity of care	23 items	5 point Likert scales on level of true/false of statements and level of agreement with statements	Study on a random sample of primary care patients demonstrated high internal reliability and better correlation with patient satisfaction compared with other continuity measures completed by providers *(no further detail of this work available).*
Generic Measure of Continuity of Care	Haggerty (2012)	Aims to assess patients’ perception of continuity of care	32 items across nine subscales: 1–3) main health care clinician (management and relational^a^ continuity) 2) clinicians or team care (team relational, manement, informational) 3) patient’s partnership in care (support to management^a^ and informational)	Dichotomous, open ended and 5 point Likert scales on level of agreement with statements	Pilot study on 556 patients with 2 rounds of testing to identify subscales (Cronbach’s alpha of subscales ranged from 0.66–0.93) and correlations with pre-identified indicators of continuity (0.65–0.78) [15].
Medical Care Questionnaire	Harley (2009)	Aims to assess patients’ experiences of continuity of care	15 items with three constructs: Communication, Coordination and preferences.	5 point Likert scales on level of agreement with statements	Pilot study on 677 oncology patients with 2 rounds of testing to identify subscales (Cronbach’s alpha of subscales ranged from 0.69–0.84) and test discriminate validity (differences in ratings between 2 groups of patients with high/low preferences of seeing the same doctor).
Nijmegen Continuity Questionnaire	Uijen (2011, 2014)	Aims to explore patients’ perspectives of the patient-provider relationship	29 items across three subscales: 1) Personal continuity (my provider knows me)^a^ 2) Personal continuity (my provider shows commitment)^a^ 3) Team/cross boundary continuity	5 point Likert scale on level of agreement with statements	2 pilot studies to identify subscales (Cronbach’s alpha of subscales ranged from 0.82–0.89), confirm construct validity against other tools, and assess test-retest reliability (ICC 0.71–0.82) [20, 40].
Patient-doctor depth of relationship	Ridd et al. (2011)	Aims to assess patient’s perceptions of the depth of relationship with their doctor	8 items with two constructs: usual provider of care/preference for care, and relationships. A score output indicates the depth of relationship between 0 (none at all) to 32 (very strong relationship).	5 point Likert scales on level of agreement with statements	Pilot study on 529 patients with 2 rounds of testing to examine face validity (via interviews), internal reliability (Cronbach’s alpha of 0.93), and test-retest reliability (ICC 0.87) [42].
Patient-doctor Relationship questionnaire-9 (PDRQ-9)	Van der Feltz-Cornelis (2004)	Aims to assess patient’s perceptions of the relationship with their doctor	9 items with no disparate subscales	5 point Likert scales on level of agreement with statements	Pilot study on 165 patients to identify subscales (only one construct identified) [39]. Also been validated in a German-speaking population [21].
Primary Care Assessment Survey (PCAS)	Safran (1996)	Aims to assess patients’ experiences of primary care	57 items via 11 summary scales to measure 7 domains of care: 1) accessibility, 2) longitudinal continuity^a^, 3) comprehensiveness, 4) integration, 5) clinical interaction, 6) interpersonal treatment^a^, 7) trust^a^.	5 point Likert scales on level of performance from ‘very poor’ to ‘excellent’	Testing data derived from 7204 patients during a 2-year study on primary care performance. Internal consistency of subscales was tested (Cronbach’s alpha ranged from 0.8–1.0). Item-convergence validity, item-discrimination validity, item variance, score reliability all tested.
Primary Care Assessment Tool (PCAT)	The John Hopkins Primary Care Policy Center for Underserved Populations	Aims to measure the extent and quality of primary care services at an individuals main source of general care	93 items via 9 subscales examining 1) Accessibility^a^ 2) Utilisation 3) Longitudinally of interpersonal relationships or ongoing care^a^ 4) Coordination of services, 5) Comprehensiveness of services 6) Comprehensiveness of care received 7) Family centeredness^a^ 8) Community orientation, and 9) Cultural experience	4 point Likert scales on level of agreement with statements	Adult version tested through a pilot study with surveys and interviews to investigate reliability, validity and scoring analyses of the 9 subscales [43]. Other validation studies have been conducted in different population groups and settings.
Primary Care Evaluation Tool (PCET)	Regional Office for Europe of WHO	Aims to assess patients’ and providers’ perspectives of good primary health care system and service delivery	45 items covering four topics: 1) Continuity^a^ 2) Accessibility 3) Comprehensiveness 4) Coordination	Unknown	Unknown *(no validation work has been published)*
Therapeutic Bond Scale	Saunders et al. (1989)	Aims to assess patients’ perspective of the quality of therapeutic relationship with their provider	50 items across three subscales: 1) working alliance^a^ 2) empathic resonance^a^ 3) mutual affirmation^a^	5 point Likert scale on level of agreement with statements	Study on 113 psychotherapy outpatients to test correlation of subscales on patients’ rating of session quality (*p* < 0.05 for all subscales) and outcome (*p* > 0.05 for all subscales) [44].
Working Alliance Inventory Short form revised (WAI-S)	Hatcher & Gillaspy (2006)	Aims to assess patients’ experiences with their therapist. Based on the full working alliance inventory.	12-items across three subscales: 1) Agreement on the tasks of therapy 2) Agreement on the goals of therapy 3) Development of an affective bond^a^	5 point Likert scale on frequency of activities from ‘never’ to ‘always’.	Pilot study on 466 patients with 2 rounds of testing to examine pre-identified subscales (subscales were not able to be confirmed) [45].
N/A	Al-Azri et al. (2014)	Aims to assess patients’ perceptions and experiences of primary care	33 items in two sections: 1) Perception of relational continuity 2) Experience/application of relational continuity	3 point Likert scale on level of agreement with statements	Pilot study on 50 patients to test suitability of tool *(no further detail of this work available)* [22]
N/A	Hansen (2016)	Aims to explore how women with chronic fatigue experience GP care	3 items exploring experiences in consultations	4 point Likert scale on frequency of activities from ‘never’ to ‘always’.	Pilot study on 143 patients to test suitability of tool *(no further detail of this work available)* [28]

^a^Indicates relevant subscales to this review

Table 3 provides information on the feasibility of using the nine remaining questionnaires. The questionnaires are diverse in length; the shortest being the Therapeutic Bond Scale (6 items; 30 s to complete) and the longest being the Care Continuity Across Levels of Care Scale (73 items, up to 15 min to complete). Some of the questionnaires focus entirely on assessing quality of relationships, such as Patient-Doctor Relationship Questionnaire (100% relevant items). However, for other questionnaires, relationship quality is not the only focus, such as the Generic Measure of Continuity Scale (34% relevant items) and Nijmegan Continuity Questionnaire (28% relevant items). Seven of the questionnaires are freely available for use, whereas the Generic Measure of Continuity Scale and Therapeutic Bond Scale requires payment prior to use. All the questionnaires are in the English language, with several also translated to other languages.

**Table 3:** Overview of questionnaires that assess quality of relationships

Questionnaire	Estimated completion time	Number of items	Number of relationship focussed items (%)	Freely available	Languages available	Additional pilot work likely required prior to use
Care Continuity Across Levels of Care Scale (CCAENA)	10–15 min	73	10 (14%)	Yes [46]	English, Spanish	No
Chao-Perception of Continuity Scale	10–15 min	23	20 (87%)	Yes [47]	English	Yes^b^
Generic Measure of Continuity Scale	15 min	32	11 (34%)	No –subscription required [15]	English, French	Yes^b^
Medical care questionnaire	< 5 min	15	4 (26%)	Yes [16]	English	Yes^b^
Nijmegen Continuity Questionnaire	15 min	29	8 (28%)	Yes [48]	English, Dutch, Norwegian	No
Patient-Doctor Depth of Relationship	< 5 min	8	7 (88%)	Yes [49]	English	No
Patient Doctor Relationship Questionnaire (PDRQ-9)	< 5 min	9	9 (100%)	Yes [39]	English	Yes^b^
Therapeutic Bond scale	30 s	6	Unknown	No – payment required [50]	English	Yes^b^
Working Alliance Inventory –Short Form revised	5 min	12	8 (66%)	Yes [51]	English, Argentinian, Chilean, Chinese, Danish, Dutch, Finish, French, German, Italian, Japanese Lithuanian, Norwegian, Polish Portuguese, Slovenian, Spanish, Urdu	Yes^b^

^a^Estimated completion time based description of questionnaire where possible, or authors’ interpretation

^b^No evidence of establishing reliability of construct validity, which may preclude its use in evaluation work

## Discussion

This study systematically reviewed the body of evidence to address the lack of understanding on how to best measure the quality of continuous relationships between patients and primary care providers. Fourteen relevant questionnaires were found in the 27 studies included in the review. Of the 14 questionnaires, nine were considered as potentially feasible for future use, including three that were considered strongest candidates based on being relevant, freely available in English and not needing additional pilot work prior to use. These three questionnaires are the Care Continuity Across Levels of Care Scale (CCAENA), the Nijmegan Continuity Questionnaire and the Patient-Doctor Depth of Relationship Tool.

The decision to use one of the reviewed questionnaires in future work requires careful consideration. Some of the questionnaires focussed solely on assessing quality of relationships and did not examine any other topics, for example the Patient Doctor Relationship Questionnaire (PDRQ-9) [39]. However, for this questionnaire, no evidence of pilot testing was found that confirmed the content was relevant and sufficiently comprehensive to assess the experiential nature of relationship quality. Questionnaires with a broader focus could be interpreted as less relevant, such as the Nijmegan Continuity questionnaire (28% relevant items), however this questionnaire has confirmed construct validity and test-retest validity, demonstrating its appropriateness for future use in research [20, 40]. Researchers and primary care workers are encouraged to consider several factors that may impact on the use of these questionnaires in their work, including their scope, focus, length, availability and validity testing.

Caution is needed when interpreting the level of validity testing undertaken for the questionnaires used in the studies in the review. Diverse terms were used to describe the same type of validity testing (such as internal consistency and construct validity) [15, 20, 31, 40]. Furthermore, only two studies assessed test-retest reliability, the Nijmegan Continuity Questionnaire and Patient-doctor depth of relationship tool. Confirming test-retest reliability is considered essential for evaluations of interventions in order to be confident that any changes seen in results over time is due to a change in service rather than natural variation of results [41]. Undertaking validity testing does not guarantee that a questionnaire is “valid”. For example, the authors of the Generic Measure of Continuity Scale conducted a pilot study to investigate its correlation with pre-identified indicators of continuity and found very low correlation [15]. No changes were made to the scale to ameliorate the low correlation, which hampers its use without further development work. Conversely, the Patient-doctor depth of relationship tool has undertaken the most comprehensive pilot testing work of all the reviewed questionnaires and has confirmed good face validity, high internal reliability and strong test-retest reliability, indicating its appropriateness for future use [42].

This is a comprehensive review which identified six questionnaires that were not captured in the previous review [12]. Two of the questionnaires were included in the previous systematic review (Patient-doctor depth of relationship tool and Patient doctor relationship questionnaire (PDRQ-9)) and continue to be used in studies [21, 36]. The remaining questionnaires have been developed or refined since this time, indicating an increasing focus on this aspect of health care evaluation. However, this review has also identified some notable limitations regarding questionnaires assessing quality of relationships between patients and primary care providers. None of the questionnaires consider providers’ perspectives relationships, or the association between patients’ and providers’ perspectives on their relationship. Furthermore, none of the studies investigated whether the quality of relationship predicted patient outcomes, warranting future work to confirm the notion that quality of relationships is associated with improved outcomes.

## Conclusions

This study provides an overview of 14 unique questionnaires that have been used to assess the quality of relationships between patients and primary care providers. This area is of increasing importance with the growing focus on patient engagement as a critical element in the prevention and management of chronic disease and unhealthy lifestyle choice. The selection of a questionnaire for future work should be based on its scope, focus, length and feasibility for use in the setting in which it will be applied.

## Abbreviations

CCAENA: Care Continuity Across Levels of Care Scale [translated from Spanish: Cuestionario Continuidad Asistencial Entre Niveles de Atención]
CINAHL: Cumulative Index of Nursing and Allied Health Literature
PDRQ-9: Patient Doctor Relationship Questionnaire – 9 item
PRIMSA: Preferred Reporting Items for Systematic Reviews and Meta-Analyses
